# A *bovine herpesvirus 1* pUL51 deletion mutant shows impaired viral growth *in vitro* and reduced virulence in rabbits

**DOI:** 10.18632/oncotarget.7771

**Published:** 2016-02-26

**Authors:** Sohail Raza, Mingliang Deng, Farzana Shahin, Kui Yang, Changmin Hu, Yingyu Chen, Huanchun Chen, Aizhen Guo

**Affiliations:** ^1^ The State Key Laboratory of Agricultural Microbiology, Huazhong Agricultural University, Wuhan, China; ^2^ College of Veterinary Medicine, Huazhong Agricultural University, Wuhan, China; ^3^ Department of Pathobiological Sciences, School of Veterinary Medicine, Louisiana State University, Baton Rouge, Louisiana, United States of America; ^4^ College of Animal Science and Technology, Huazhong Agricultural University, Wuhan, China; ^5^ Key Laboratory of Development of Veterinary Diagnostic Products, Ministry of Agriculture, Wuhan, China; ^6^ International Joint Research and Training Centre for Veterinary Epidemiology, Hubei Province, Wuhan, China

**Keywords:** bovine herpesvirus 1, bacterial artificial chromosome, morphogenesis, pUL51, tegument protein, Immunology and Microbiology Section, Immune response, Immunity

## Abstract

*Bovine herpesvirus 1* (BoHV-1) UL51 protein (pUL51) is a tegument protein of BoHV-1 whose function is currently unknown. Here, we aimed to illustrate the specific role of pUL51 in virion morphogenesis and its importance in BoHV-1 virulence. To do so, we constructed a BoHV-1 bacterial artificial chromosome (BAC). We used recombinant BAC and transgenic techniques to delete a major part of the UL51 open reading frame. Deletion of pUL51 resulted in severe viral growth defects, as evidenced by lower single and multi-step growth kinetics, reduced plaque size, and the accumulation of non-enveloped capsids in the cytoplasm of infected cells. Using tagged BoHV-1 recombinant viruses, it was determined that the pUL51 protein completely co-localized with the *cis*-Golgi marker protein GM-130. Taken altogether, pUL51 was demonstrated to play a critical role in BoHV-1 growth and it is involved in viral maturation and egress. Moreover, an *in vivo* analysis showed that the pUL51 mutant exhibited reduced virulence in rabbits, with no clinical signs, no nasal shedding of the virus, and no detectable serum neutralizing antibodies. Therefore, we conclude that the BoHV-1 pUL51 is indispensable for efficient viral growth *in vitro* and is essential for virulence *in vivo*.

## INTRODUCTION

*Bovine herpesvirus 1* (BoHV-1) is an important pathogen that causes pneumonia, conjunctivitis, genital disorders, and abortions in cattle. Additionally, BoHV-1 is an important factor in shipping fever [1, 2]. As a result, BoHV-1 causes significant economic losses to the cattle industry [3]. BoHV-1 is a member of the *Alphaherpesvirinae* subfamily. The mature BoHV-1 virion consists of defined structures that are found in all herpesviruses: the nucleocapsid, tegument, and envelope. The tegument proteins function to establish suitable conditions for efficient viral growth, assembly, and egress [4]. Virion assembly and egress proceed from the nucleus to the cytoplasm. These processes are explained by an envelopment, de-envelopment, and re-envelopment model. Tegument proteins play important roles at each point of virion assembly and egress [5, 6]. Herpesvirus assembly and egress is a complex and dynamic process that requires many protein interactions. As in *herpes simplex virus 1* (HSV-1), the pUL31 and pUL34 proteins play roles in primary envelopment [7, 8]; however, the tegument proteins of BoHV-1 have been poorly characterized [4].

A sequence analysis showed that the BoHV-1 pUL51 is a protein that is conserved in all herpesvirus family members. BoHV-1 pUL51 is a 243-amino acid tegument protein whose function is unknown [9]. Homologs of pUL51 include pUL71 in human cytomegalovirus (HCMV), BSRF1 in Epstein-Barr virus, and ORF55 in Kaposi's sarcoma-associated herpesvirus (KSHV) [10]. HSV-1 UL51 belongs to viral genes of the γ2 class, while BoHV-1 pUL51 belongs to the γ1 class [9]. Previous analyses of different herpesviruses showed that pUL51 is dispensable for virus growth in cell culture [11–13]. Previously, it was reported that HSV-1 pUL51 mutant affects the egress of nucleocapsids, as well as the maturation of cytoplasmic capsids [12], while a pUL51 deletion mutant of the *pseudorabies virus* (PRV), another member of the *Alphaherpesvirinae* subfamily, exhibited similar growth defects with less efficient secondary envelopment [13]. In HSV-1, pUL51 plays a role in cell-to-cell spreading by interacting with gE protein [14]. However studies showed that HCMV pUL71 affects late envelopment and viral egress [11, 15]. Nevertheless, the phenotype of a BoHV-1 pUL51 mutant has not been characterized in cell culture or *in vivo*.

In this study, we constructed and characterized a mutant BoHV-1 virus that does not express pUL51. We added an epitope tag to the capsid protein UL35 in UL51 deletion and intact UL51 backgrounds to track and explore viral assembly and egress in real time. Meanwhile, an immunofluorescence assay was performed to find localization of pUL51. The function of pUL51 in viral morphogenesis was further characterized using electron microscopy. The data demonstrated that pUL51 is involved in the efficient production of extracellular virions *in vitro* and is essential for virulence *in vivo*.

## RESULTS

### Generation of a BoHV-1 BAC plasmid

**Figure 1:**
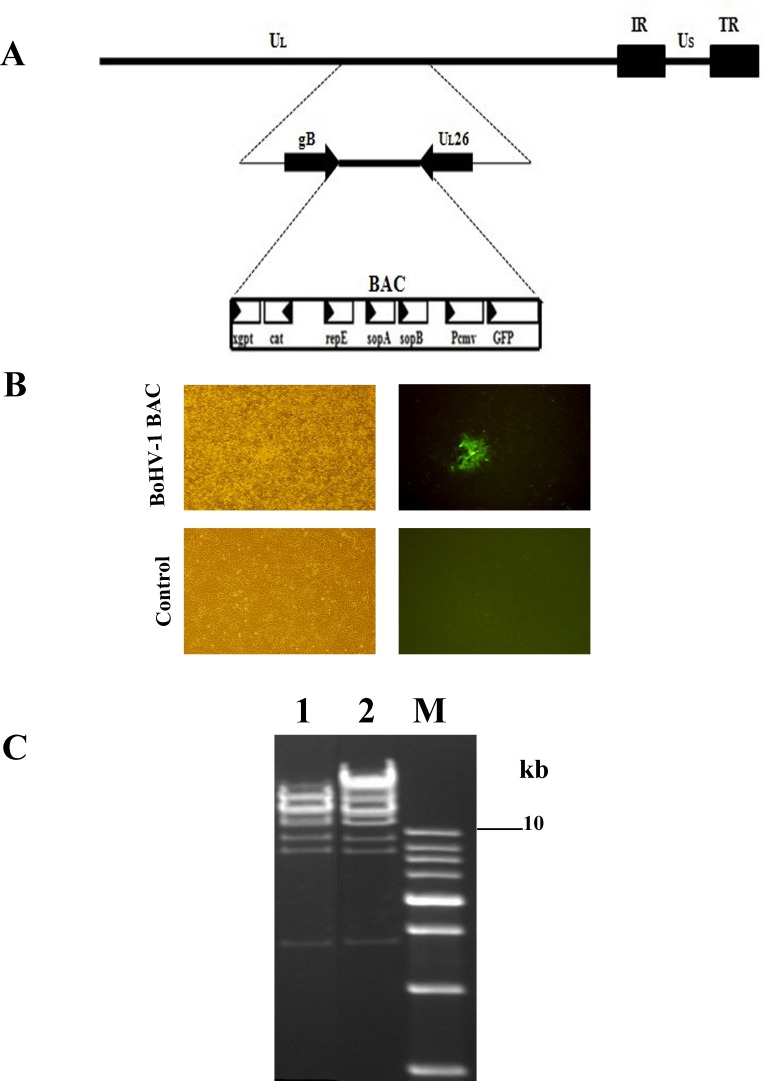
**A.** Schematic diagram showing the incorporation of a BAC plasmid within the intergenic region between the gB and UL26 genes of BoHV-1. **B.** Green fluorescence produced after co-transfection of pMDgB-BAC-UL26 with the wild-type BoHV-1 genome in MDBK cells and control cells. **C.** BoHV-1 recombinant BAC clones were picked for RFLP analysis. Lane 1, restriction analysis shows a wild-type BoHV-1, while lane 2 shows a BoHV-1 recombinant BAC clone.

To construct a BoHV-1 bacterial artificial chromosome (BAC), first we inserted BAC plasmid sequence within intergenic region of gB-UL26. Then Mardin-Darby bovine kidney (MDBK) cells in a confluent monolayer were transfected with the pMDgB-BAC-UL26 plasmid and the wild-type BoHV-1 genome (Figure 1A). Recombinant BoHV-1 containing the BAC emitted green fluorescence (Figure 1B). A circular, viral DNA of BoHV-1 BAC was isolated and electroporated into *Escherichia choli* cells. A BoHV-1 BAC-positive clone was selected by chloramphenicol and confirmed by restriction fragment length polymorphism (RFLP) analysis using the HindIII restriction enzyme (Figure 1C). To reconstitute the virus, the selected pBoHV1-BAC DNA was co-transfected with pCAGGS-NLS/Cre into MDBK cells using the calcium phosphate method, and the resultant virus was named vBoHV-1 (wild-type).

### Construction and characterization of recombinant viruses

**Figure 2:**
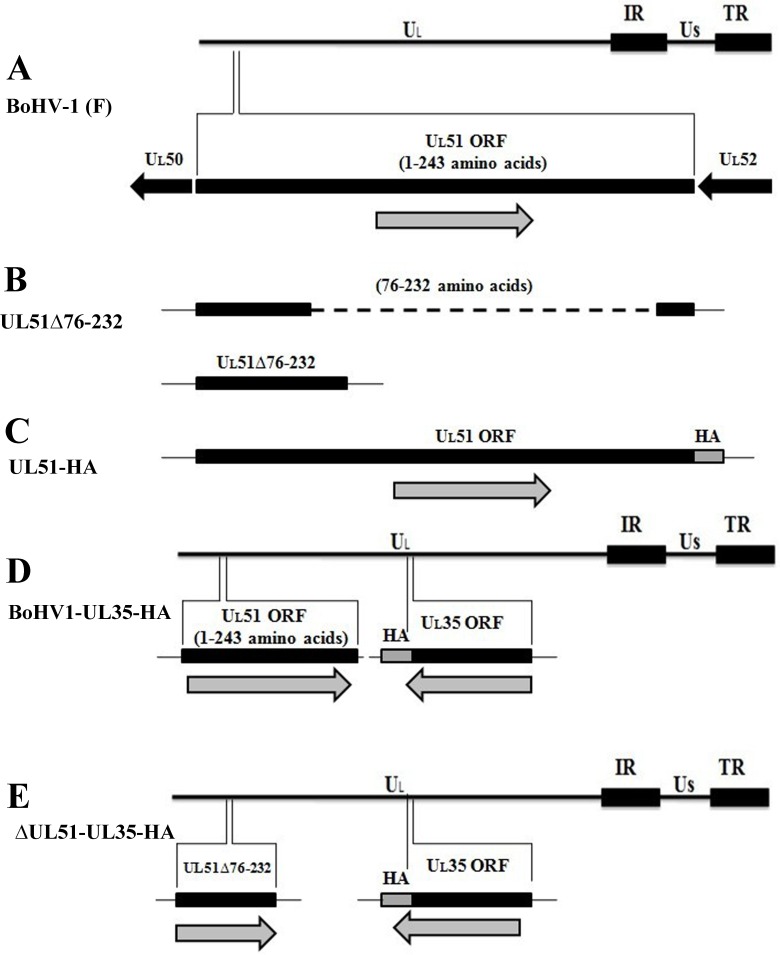
Schematic illustration and characterization of the recombinant viruses used in this study **A.** Diagram showing the entire BoHV-1 genome and its components, along with the position of the UL51 gene in the BoHV-1 genome. **B.** Diagram showing the mutation of UL51 by deleting amino acids 76-232 (Dash line showed the deleted region of UL51). **C.** The insertion of an HA tag at the carboxyl terminus of the UL51 protein. **D.** Schematic diagram showing the insertion of an HA tag at the carboxyl terminus of the UL35 protein in the wild-type BoHV-1 BAC. **E.** The insertion of an HA tag at the carboxyl terminus of the UL35 protein in the BoHV1-UL51Δ76-232 virus.

**Figure 3:**
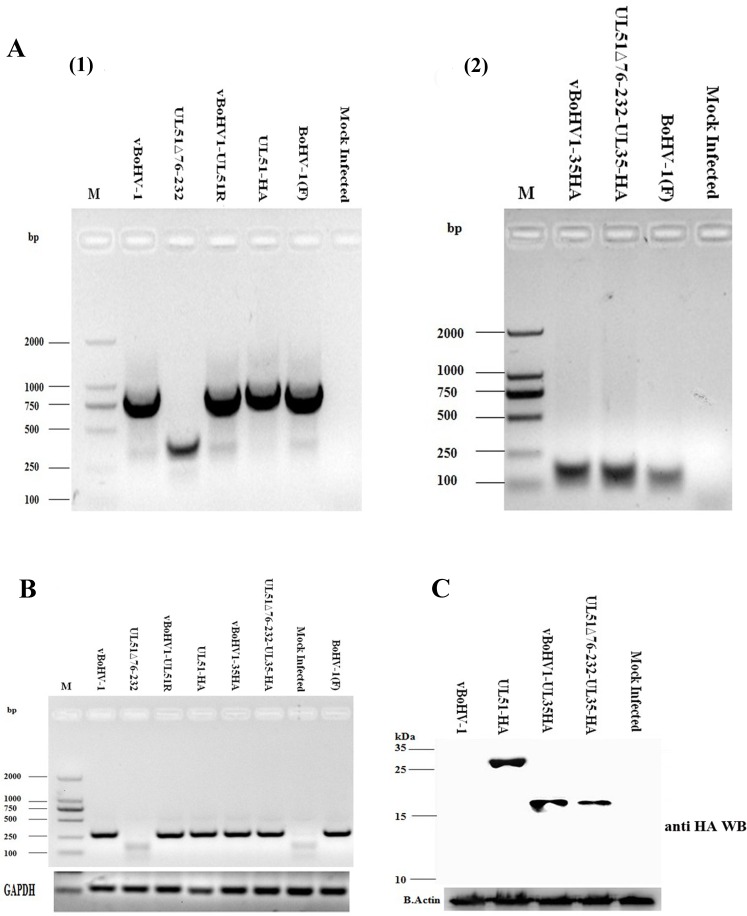
Confirmation of recombinant viruses **A.** Viral DNA was extracted using the TIANamp Virus DNA/RNA kit (Tiangen Biotech, Beijing, China) from MDBK cells infected with the indicated viruses at an MOI of 3. PCR were run with the indicated viral DNAs of pUL51 using the UL51 F/R primers (Table 2) and pUL35 using the UL35 F/R primers (Table 2). **B.** Expression of pUL51 by the recombinant viruses. RT-PCR was performed with the indicated viral cDNAs using the pUL51Exp. F/R primers (Table 2) and the PCR products were analyzed by agarose gel electrophoresis. The bovine glyceraldehyde 3-phosphate dehydrogenase (GAPDH) gene was used as an internal reference control. **C.** Expression of HA-tagged recombinant viruses. MDBK cells were infected with the indicated viruses and lysed at 18 hpi. Proteins were probed by Western blotting using an anti-HA antibody. Beta-actin was used as an internal control.

Figure 2A shows the whole genome map of BoHV-1 and the position of UL51 gene. A two-step, λ-red-mediated mutagenesis/”en passant” mutagenesis protocol was used to successfully construct several mutant viruses, including the UL51Δ76-232 (UL51 mutant) (Figure 2B), UL51R (UL51 revertant), vBoHV1-UL51HA (HA-tagged UL51) (Figure 2C), vBoHV1-UL35HA (HA-tagged UL35) (Figure 2D), and vBoHV1-ΔUL51-UL35HA (UL51 mutant and HA-tagged UL35) viruses (Figure 2E). The mutation and reversion of the mutation in the UL51 gene were confirmed by polymerase chain reaction (PCR) and DNA sequencing (Figure 3A). To determine the properties of pUL51, an HA tag was attached to the carboxyl terminus of UL51, and the resulting virus was named the vBoHV1-UL51HA virus. To analyze changes in the assembly and egress of virions in the UL51 mutant virus, an HA tag was added to the carboxyl terminus of UL35 in the pBoHV-1 BAC (wild-type) and pBoHV1-UL51Δ76-232 BAC (UL51 mutant) viruses. The addition of the HA tags in the vBoHV1-UL51HA, vBoHV1-UL35HA, and vBoHV1-ΔUL51-UL35HA viruses was confirmed by PCR and DNA sequencing (Figure 3A). To ensure that the region of UL51 was deleted, we performed reverse transcription PCR (RT-PCR), and as shown in Figure 3B, pUL51 was not expressed in the UL51Δ76-232, virus while all of the other recombinant viruses produced high levels of pUL51 mRNA (Figure 3B). The expression of the HA-tagged pUL51 protein in these recombinant viruses was further confirmed by western blotting (Figure 3C).

### Analysis of viral growth

**Figure 4:**
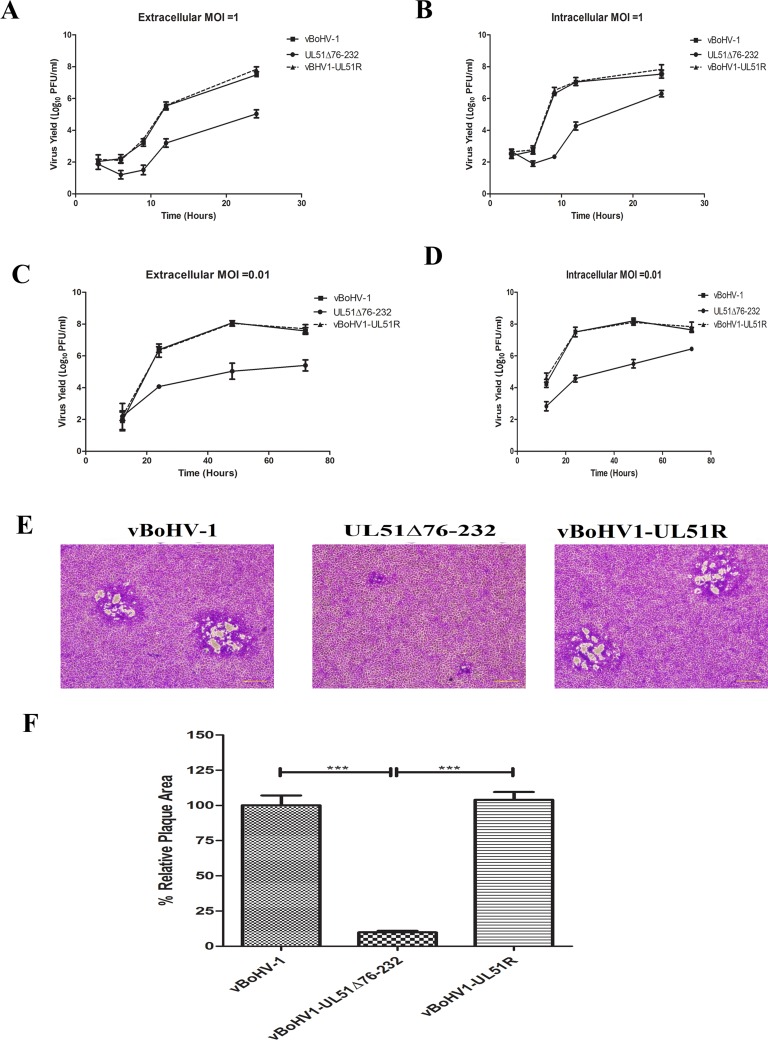
Growth kinetics of the UL51 mutant virus in MDBK cells Growth of the wild-type (■), UL51Δ76-232 mutant (●) and UL51 revertant (▲) viruses. For single-step growth kinetics, MDBK cells were infected with an MOI of 1. At 3, 6, 9, 12, and 24 hpi, extracellular **A.** and intracellular **B.** virus particles were collected, and virus yields were determined by titration on MDBK cells. Each data point represents the mean ± standard deviation of three independent experiments. For multi-step growth kinetics, MDBK cells were infected with the indicated viruses at an MOI 0.01. Extracellular **C.** and intracellular **D.** virus particles were sampled at 12, 24, 48, and 72 hpi, and titrated on MDBK cells. Each data point represents the mean ± standard deviation of three independent experiments. **E.** Plaque morphology and plaque size of the vBoHV-1, UL51Δ76-232, and vBoHV1-UL51R viruses. MDBK cells were infected with 200 PFU per well of the indicated viruses, and overlaid with methylcellulose. After 48 h, the cells were fixed and stained with crystal violet. **F.** The plaque area was analyzed with an Olympus IX70^®^ Microscope. The mean percentages of the plaques and standard errors were determined by counting 50 random plaques of each virus, and the significance level was calculated using a *t*-test (***, *P* < 0.0001).

It has been reported that pUL51 is not essential for viral replication [16]. To analyze the effect of pUL51 on viral growth in detail, the growth of the vBoHV1-UL51Δ76-232 (the UL51 mutant) virus was investigated. First, we studied the growth kinetics of wild-type, UL51 mutant, and vBoHV1-UL51R (revertant) viruses. Single-step growth kinetics (Figure 4A, 4B) showed a considerable reduction of the extracellular and intracellular virus titers in the UL51 mutant virus compared with those of the other viruses. Surprisingly, the intracellular titer of UL51 mutant virus was ∼1 log higher than that its extracellular titer. Multi-step growth kinetics (Figures. 4C, 4D) yielded similar results. These results suggest that pUL51 may decrease the cytoplasmic exit of virions.

To examine the effect of the UL51 mutation on cell-to-cell spreading, the plaques formed by each virus were examined, and the diameters of 50 plaques of each virus were measured at 48 h post-infection (hpi). The size and morphology of the plaques formed by the UL51 mutant virus were distinct from those of the wild-type and revertant viruses (Figure 4E). The UL51 deletion mutant virus produced (*P* < 0.0001) smaller plaques than the wild-type and revertant viruses (Figure 4F); the plaque size of the UL51 mutant was approximately 10% of that of the wild-type virus.

### Characterization of the UL51 protein

**Figure 5:**
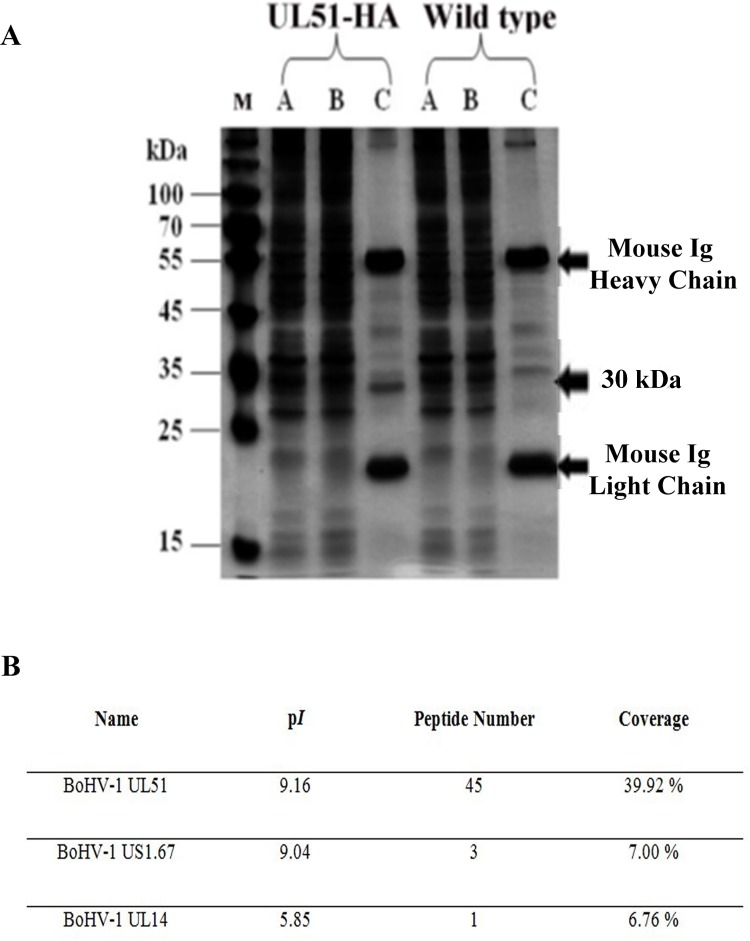
Characterization of the pUL51 protein **A.** Analysis of precipitated pUL51 protein using anti-HA antibody-conjugated magnetic agarose beads. The cellular lysate (lane A), flow-through (lane B), and elute (lane C) were separated on SDS-PAGE, and after silver staining, an expected 30-kDa band corresponding to pUL51 was found. **B.** Mass spectrometry analysis of proteins immunoprecipitated with anti-HA agarose and the protein present in the gel corresponding to the proteins, of which molecular weight were approximately 30 kDa were identified by mass spectrometry.

To characterize the UL51 protein, MDBK cells were infected with the vBoHV1-UL51HA virus and a control wild-type virus. After 18 h, the cells were lysed, and the lysate was incubated with anti-HA-tag monoclonal antibody-conjugated magnetic agarose beads. Bound proteins were collected and analyzed by sodium dodecyl sulfate-polyacylamide gel electrophoresis (SDS-PAGE). Silver staining revealed that the vBoHV1-UL51HA sample contained an additional band of the expected size compared with the wild-type sample (Figure 5A). The identity of the corresponding protein, as well as any post-translational modifications, was determined using matrix-assisted laser desorption/ionization time-of-flight (MALDI-TOF) mass spectrometry. The mass spectrometry analysis confirmed that the indicated band was the BoHV-1 UL51 protein (Figure 5B). Furthermore, a post-translational modification analysis predicted that amino acid residue Ser31 was phosphorylated. The original size of the UL51 proteinis 28 kDa, while the size of the HA tag is 1 kDa and the phosphorylation modification added more than 1 kDa. The total size of pUL51 was approximately 30 kDa, which indicates that the 1-kDa size difference may be due to the phosphorylation of the UL51 protein.

### Intracellular co-localization of the UL51 protein with cis-Golgi marker protein (GM-130)

**Figure 6:**
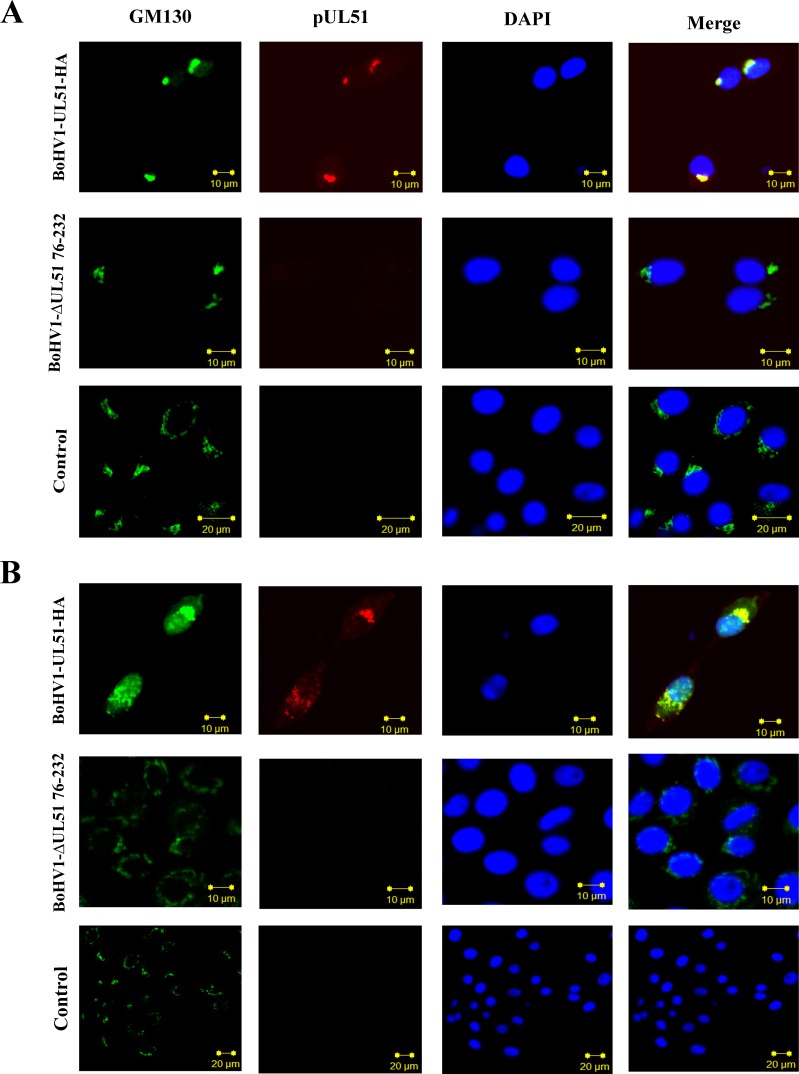
Intracellular co-localization of pUL51 with the *cis*-Golgi marker GM130 using laser-scanning confocal microscopy MDBK cells infected with the vBoHV-1UL51-HA and UL51 mutant viruses were fixed for immunofluorescence at **A.** 12 hrs and **B.** 18 hrs post infection. UL51-HA in the cells was stained red by a mouse anti-HA antibody and Cy3-conjugated goat anti-mouse IgG; the Golgi was stained green by a rabbit anti-GM130 primary antibody and FITC-conjugated goat anti-rabbit IgG. The cellular DNA was stained blue with DAPI. The stained cells were observed under a Zeiss LSM 510 laser-scanning confocal microscope.

Previously, it was reported that HSV-1 pUL51 co-localizes with Golgi marker proteins in the absence of other viral proteins; however, in virus-infected cells, this protein only partially co-localizes with Golgi markers [17]. To examine the localization of BoHV-1 pUL51 *in situ*, we performed an indirect immunofluorescence assay in cells infected with the vBoHV1-UL51HA virus. Interestingly, the localization of pUL51 differed from the previous findings. Confocal microscopy images showed that in BoHV1-UL51-HA virus infected cells, the pUL51 completely co-localized with the *cis*-Golgi marker protein GM130 at 12 and 48 hpi (Figure 6). As expected, no pUL51signal of was detected in UL51 mutant and control cells. Therefore, this experiment shows that BoHV-1 pUL51 may associated with maturation of virions.

### The effect of the UL51 mutation on the virion egress pathway

**Figure 7:**
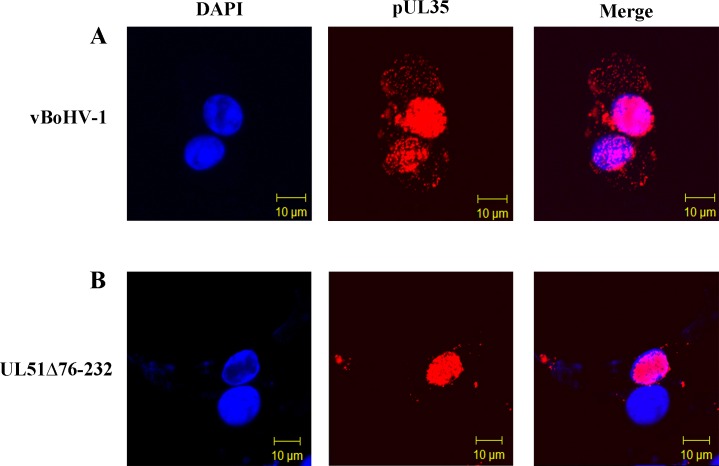
Effect of the pUL51 mutation on capsid movement MDBK cells were infected with the indicated viruses expressing HA-tagged UL35 at an MOI of 3 (wild-type virus, panel **A.** UL51Δ76-232 virus, panel **B.**). At 18 hpi, the cells were fixed and stained with a primary antibody (mouse anti-HA) and then with Cy3-conjugated goat anti-mouse IgG. DNA was stained with DAPI, and the cells were observed under a Zeiss LSM 510 laser-scanning confocal microscope.

To examine the effect of the UL51 mutation on capsid assembly and egress *in situ*, an HA tag was attached to the pUL35 protein in the wild-type (pBoHV1-BAC) and UL51 mutant (pBoHV1-UL51Δ76-232-BAC) viruses. Freshly grown MDBK cells on glass coverslips were infected with HA-tagged virus bearing intact pUL51 (vBoHV1-UL35HA) or the UL51 deletion (vBoHV1-UL51Δ76-232-UL35HA) at a multiplicity of infection (MOI) of 3 plaque-forming units (PFU) per cell. As shown in Figure 7, at 18 hpi, there was a high density of concentrated, punctate fluorescence in cells infected with the wild-type virus (Figure 7A) compared with those infected with UL51 mutant (Figure 7B). Meanwhile, the mutant pUL35 protein localized in the cytoplasm (Figure 7B) and at the edges of the cells (Figure 7B) compared with the wild-type pUL35 protein that distributed throughout cytoplasm (Figure 7A). These images showed that the UL51 mutation may impair the nuclear egress of capsids, which is similar to a previous finding for HSV UL51 [12].

### Transmission electron microscopy (TEM)

**Figure 8:**
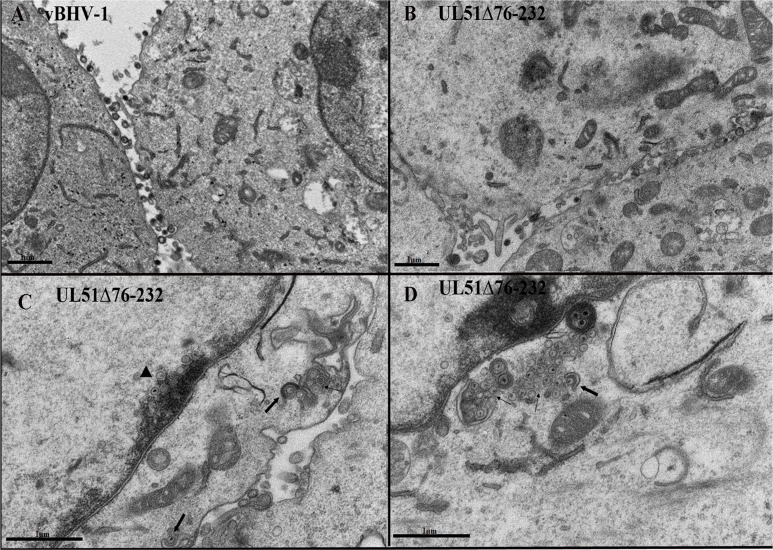
TEM analysis of the UL51 mutant virus MDBK cells were infected with the vBoHV-1 **A.** and vBoHV1-UL51Δ7-232 viruses **B. C.** and **D.**, and at 18 hpi, the cells were processed for TEM. In UL51 mutant-infected cells, mature, intracellular and extracellular virions were rarely seen **B. C.** and **D.** compared with wild-type virus-infected cells. In UL51 mutant-infected cells, **C.** a large number of nucleocapsids accumulated near the nuclear membrane (arrowheads). Aggregates of cytoplasmic capsids without secondary envelopment were found in UL51 mutant-infected cells (C. D. thin arrows). Impaired secondary envelopment was observed in mutant virus-infected cells **C.** and **D.**, thick arrows).

To further confirm the role of pUL51 in BoHV-1 morphogenesis, we examined virus particles using TEM. MDBK cells were infected with the UL51 mutant virus or the wild-type virus as a control. At 18 hpi, the cells were processed for TEM. Few extracellular mature virions were observed on the cell membrane of UL51 mutant-infected cells; while great numbers of extracellular virions were observed on the outer surface of wild-type virus-infected cells (Figure 8A, 8B). As predicted from the immunofluorescence experiments, a huge number of nucleocapsids were present on the inner side of the nuclear membrane of UL51 mutant-infected cells (Figure 8C), while this type of nucleocapsid aggregation was not observed in control cells infected with the wild-type virus. This morphometric analysis supports our previous finding that the UL51 mutation may decrease the nuclear egress of the nucleocapsids. Furthermore, in UL51 mutant-infected cells, only a few mature virions were seen in the cytoplasm, while most of the cytoplasmic capsids remained non-enveloped or partially enveloped (Figures 8C, 8D), and there were fewer mature virions near the plasma membrane, in preparation for egress, in UL51 mutant-infected cells.

**Figure 9:**
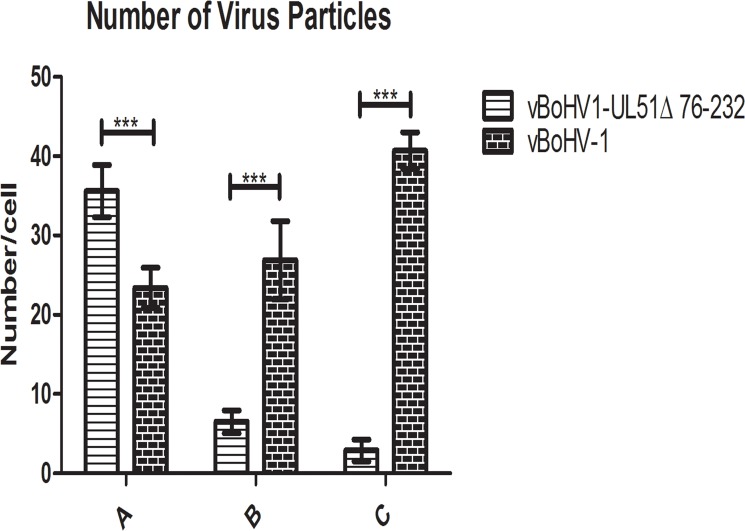
Quantitative analysis of virion distributions in cellular compartments based on TEM observations MDBK cells were infected with the vBoHV-1 or UL51Δ76-232 viruses at an MOI of 3. At 18 hpi, the samples were processed for TEM. The number of virus particles in each compartment of 40 infected cells was counted for each sample. Each bar represents the mean ± standard deviation, and a two-way ANOVA was used to determine statistically significant differences (***, *P < 0.001*). **A.** Nucleocapsids in the nucleus; **B.** mature virions in the cytoplasm; C. extracellular, mature virions.

To illustrate the effect of the UL51 mutation, we quantified the number of virus particles in different cellular compartments. We counted 40 cells randomly in more than 25 fields each of vBoHV-1- and UL51Δ76-232-infected cells. In UL51 mutant-infected cells, there was a marked increase in the number of nucleocapsids, while fewer mature virions were found in the cytoplasm. Additionally, there were fewer extracellular, mature virions in UL51 mutant-infected cells than in wild-type virus-infected cells (Figure 9). The above ultrastructural analyses indicate that pUL51 plays an important role in nuclear egress, secondary envelopment and cytoplasmic exit of the virions

### Properties of vBoHV1-UL51Δ76-232, vBoHV1, and vBoHV1-UL51R viruses in rabbits

**Figure 10:**
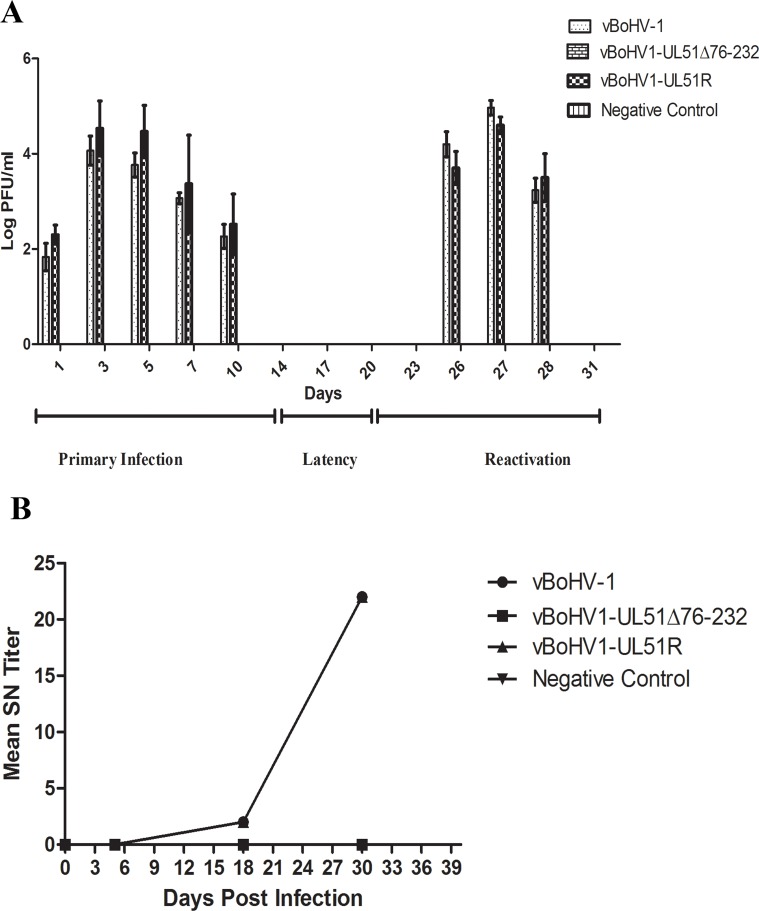
Rabbit responses after infection with different BoHV-1 viruses with intact, revertant, and deleted pUL51 **A.** Nasal virus shedding: nasal swabs of rabbits infected with the vBoHV-1, vBoHV1-UL51Δ76-232, and vBoHV1-51R viruses, as well as a negative control, were collected at the indicated time points and titrated in MDBK cells. A bar represents the mean ± standard deviation of each virus. **B.** SN antibody production. Mean SN antibody titers were calculated for the indicated groups at different time points. Each data point represents the mean value of the individual group.

To analyze the phenotype of the UL51 mutant virus *in vivo*, rabbits were infected intranasally with the indicated numbers of PFU of the wild-type, UL51 mutant, and revertant viruses, and nasal swabs were collected and analyzed for viral shedding during the primary, latent, and reactivation phases of the infection. In the revertant and wild-type virus infections, during the primary phase, the nasal shedding of virus started at 1 d post-infection (dpi), peaked at 3 dpi, and continued until 10 dpi (Figure 10A), while there was no viral shedding in the UL51 mutant-infected and blank control groups. During the latent phase, no viral shedding was observed in any of the groups. After reactivation by dexamethasone injection, there was high nasal shedding of the virus from the groups infected with the wild-type or revertant viruses, while no nasal shedding of the virus was observed in the UL51 mutant-infected and blank control groups. No animals in any group died during the infections (Table 1). These results show that in the absence of pUL51, viral growth was severely compromised and attenuated *in vivo*.

**Table 1:** Virus recovery and clinical signs from infected rabbits

Groups	Infected Virus	Infection dose per rabbit (PFU)	Nasal discharge (No.)	Virus recovered during Primary Infection (No.)	Virus recovered after Dexamethasone treatment (No.)	No. of death (No.)
1	vBHV-1	2×10^7^	6/6	6/6	6/6	0/0
2	vBHV1-UL51Δ76-232	2×10^7^	0/6	0/6	0/6	0/0
3	vBHV1-UL51R	2×10^7^	6/6	6/6	6/6	0/0
4	Blank Control	2×10^7^	0/6	0/6	0/6	0/0

### Titers of serum neutralizing (SN) antibodies of infected rabbits

Sera collected from infected rabbits were processed for SN antibody titers. At day 5 of the primary infection stage, no group produced measureable levels of SN antibodies. During the latent phase of infection, rabbits infected with the vBoHV1-UL51R or vBoHV1 viruses produced an average SN antibody titer of 3, but after reactivation of the virus, the SN antibody titer of both groups reached 22, while no SN antibodies were detected in the vBoHV1-UL51 and control groups during all phases of viral growth (Figure 10B). These results demonstrate that the UL51 mutant virus failed to induce an immune response.

## DISCUSSION

The functions of many BoHV-1 proteins during viral replication are currently unknown. We aimed to gain insights about the role of pUL51 during BoHV-1 infection. Using BAC DNA mutagenesis, we constructed a BAC containing the BoHV-1 genome. This BoHV-1 BAC showed wild-type phenotypes *in vivo* and *in vitro*. Therefore, we used this BoHV-1 BAC to construct a pUL51 mutant virus (BoHV1-UL51Δ76-232) and further showed that the pUL51 tegument protein is non-essential, but very important, for growth of the virus in cell culture, as this mutant exhibited severe growth defects and smaller plaques compared with the wild-type and revertant viruses. The small plaque phenotype and impaired viral growth have also been observed and described for all mutants of pUL51 in HSV-1 [12], PRV [13], and HCMV [11]. During the single-step and multi-step growth kinetics, in addition to the lower titer of the mutant virus, surprisingly, the intracellular titer of the UL51 mutant virus was approximately 1 log higher than that of its extracellular titer. A similar finding was also observed during the growth of a pUL71 mutant HCMV virus [11]. These agreements demonstrate that our findings are reliable. The reason for this high intracellular titer might be that that the pUL51 mutation impaired the cytoplasmic exit of viruses.

To investigate the potential role of pUL51 in viral assembly and egress, we added an epitope tag to the minor capsid protein UL35 in the pUL51 mutant and wild-type viruses. Immunofluorescence image analysis showed that in vBoHV-1 infected cells, more capsids move from nucleus to cytoplasm than in UL51Δ76-232 infected cells, and the capsids present in the cytoplasm of UL51Δ76-232 infected cell formed aggregates, which were located near the cell membrane. Similar findings were reported in HSV-1, indicating that pUL51 is involved in nuclear egress [12]. Previous work on a pUL51 homolog in HCMV also reported that it has a role in the efficient release of the virus[15].

We further hypothesized that the growth defects of the pUL51 mutant virus were related to its role in the egress pathway. To address this, we performed an ultrastructural analysis of UL51 mutant-infected MDBK cells using TEM. Electron micrographs taken at 18 hpi showed defects in nucleocapsid egress. There was a significant number of nucleocapsid aggregates on the inner side of the nuclear membrane in pUL51 mutant-infected cells, while this phenotype was not observed in any wild-type virus-infected cells. A similar nucleocapsid morphology was observed in nuclei when cells were infected with HSV-1 UL48, UL20, and UL51 mutants [18, 19]. In the present study, mature, extracellular virions were rarely visible in pUL51 mutant-infected cells. In pUL51 mutant-infected cells, there were more numbers of cytoplasmic capsids without secondary envelopment or incomplete secondary envelopment, which may lead to lower viral titers. In a previous study of PRV pUL51, the pUL51 is involved in virion morphogenesis *via* the secondary envelopment of capsids in the cytoplasm, as is PRV pUL11 [13]. Moreover, HCMV pUL71 is also involved in the late envelopment of cytoplasmic capsids, as demonstrated by its effects on multivesicular bodies [11]. Taken together, it is recognized in this study that BoHV-1 pUL51 is involved in virion morphogenesis, and that it functions in nuclear egress and the secondary envelopment of cytoplasmic capsids.

In a previous study, the HSV-1 pUL51 protein was found to co-localize with Golgi marker proteins in the absence of other viral proteins, while during infection, pUL51 partially co-localized with Golgi marker proteins [17]. Here, we showed that BoHV-1 pUL51 fully co-localized with the *cis*-Golgi marker GM130 during the early (12 h) and late phases (18 h) of infection. Because GM130 is a peripheral cytoplasmic protein that is tightly bound to the cytoplasmic end of the *cis*-Golgi matrix [20], which plays an important role in the transport of vesicles from the *cis* to *trans* end of the Golgi apparatus [21], our results demonstrate an important role of pUL51 localization at the *cis*-Golgi for the secondary envelopment of the BoHV-1 capsid. Evidence shows that Golgi-derived vesicles play an important role in secondary envelopment during the egress of virus particles [22, 23].

Following intranasal infection of rabbits, the pUL51 mutant virus was unable to propagate and incapable of causing an infection. However, the wild-type and revertant viruses exhibited a wild-type level of infection. There was no nasal shedding of virus in UL51 mutant-infected rabbits, while wild-type and revertant virus-infected rabbits started to shed virus at 1 dpi. We also detected viruses from the nasal swabs of wild-type and revertant virus-infected rabbits during the reactivation phase. The nasal swab titers of the wild-type and revertant viruses during the primary and reactivation phases corresponded to those found in another study of BoHV-1 [24]. There was a considerable amount of SN antibodies in the serum of the wild-type and revertant virus-infected rabbits, while no SN antibodies were found in UL51 mutant-infected rabbits. Thus, the absence of SN antibodies confirms that the UL51 mutant virus is unable to cause an infection *in vivo*.

Taken together, this study demonstrated that BoHV-1 pUL51 functions in virion morphogenesis and virulence, as the mutated virus showed severe growth defects in cell culture and was attenuated in a rabbit model of infection. The underlying mechanism includes impaired nuclear egress, secondary envelopment and cytoplasmic exit of the virus.

## MATERIALS AND METHODS

### Animal experiments

Animal experiment protocols were approved by the China Hubei Province Science and Technology Department, which is responsible for animal ethics in animal experiments in Hubei, China (permit no. SYXK(ER) 2010-0029), in accordance with the recommendations in the China Regulations for the Administration of Affairs Concerning Experimental Animals (1988) and the Hubei Regulations for the Administration of Affairs Concerning Experimental Animals (2005). The animal use in this study was supervised by the Committee of Experimental Animal Ethics of Huazhong Agricultural University.

### Virus strain and cell line

The BoHV-1 strain named IBRV HB06 and stored as no. CCTCC V201024 in the Tissue Culture Collection Center of China at Wuhan University was used as the parent strain in this study. BoHV-1 and its derivatives were propagated and titrated in line MDBK cells as described previously [25].

### Construction of a BoHV-1 BAC clone

The BoHV-1 BAC was constructed according to a previously described method [24]. Briefly, a BAC insertion plasmid containing a PacI insertion site within the gB-UL26 intergenic region (GenBank accession number AJ004801, nucleotides 58360 to 58366) and flanked by the upstream gB gene (1 kb) and downstream UL26 gene (1 kb) was cloned into the pMD18T- vector (TaKaRa, Dalian, China) using the primers described in Table 2; the resulting plasmid was designated pMD-gB-UL26. The mini-F (BAC) vector pHA2 containing the green fluorescent protein-encoding *gfp* gene was provided by Dr. Martin Messerle [26] and inserted into pMD-gB-UL26 at the PacI site. The resulting plasmid was designated pMDgB-BAC-UL26, and it contains the BAC plasmid sequence flanked by homologous fragments of the gB and UL26 genes (Figure 1A).

**Table 2:** Primer Sequences used in this study

Primer name	Sequence (5′ to 3′)
gB homology F	CGAGGAATTCAAGCTTGATGCGCGCGCCCGGC
gB homology R	TTCCTTAATTAAGGGGCGCCCTGCCGTGC
UL26 homology F	TTCCTTAATTAAGTTTGGCGCGCGGTGG
UL26 homology R	TACCAAGCTTGTGCGGCCTCGGCGCAC
UL51Δ (76-232aa) F	gacggcacgcggcggcttgccaaggcgcagtcgctagcgcgggcttcttcccccgccgttttgtagggataacagggtaatcgattt
UL51Δ (76-232aa) R	accgggctacgccgcaagctgcaaaacggcgggggaagaagcccgcgctagcgactgcgccttgccagtgttacaaccaattaaCC
UL51-HA F	gcggccgccgcttcttcccccgccgttttgcagcttgcggcgTACCCATACGACGTCCCAGACTACGCTtagcccggtgcaatatagggataacagggtaatc gattt
UL51-HA R	gtgttttgcgtatacttattttgcttttattgcaccgggctaAGCGTAGTCTGGGACGTCGTATGGGTAgccagtgttacaaccaattaaCC
pCDNA4 51R F	GCGGATCCTCGGCAGACGGCACGC
pCDNA4 51R R	CGGATATCCGCCGCAAGCTGCAAAACGG
pCDNA4 51R kena F	TTTTCTGCAGAGGCGGCCGCCACAAAACCACCTAGCCGCGCGGGGCGCGCGGCGGCCGTAGGGATAACAGGGT AATCGATTT
pCDNA4 51R kena R	TTTTCTGCAGGCCAGTGTTACAACCAATTAACC
UL51R Kena UL51 F	CGTCAGCGCGCTGCTGCCGGCGCCGCTGACAGTGGAGGACGTGGCGCGCTCGGCAGACGGCACGCGGCGGCT TGCCAAG
UL51R Kena UL51 R	CCACACACGTGTTTTGCGTATACTTATTTTGCTTTTATTGCACCGGGCTACGCCGCAAGCTGCAAAACGGCGGGG
UL35-HA F	ATACGAGGAGGACGCTGGCGCGGCGGCGTCTGCCGCGCGCACGTACCCATACGACGTCCCAGACTACGCTTGAC GCTTTTTTCGGTAGGGATAACAGGGTAATCGATTT
UL35-HA R	ATTATTGCGATCGCACAATTGCGCCGATCCGAAAAAAGCGTCAAGCGTAGTCTGGGACGTCGTATGGGTAGCCAG TGTTACAACCAATTAACC
UL48 F	CGGGATCCGTTGTCTTTGGGATGAGCGGGCGCA
UL48 R	CCGAATTCTAGAAGTCCAGCAGCTGGTTGAGGC
UL51 F	GACCGCGAGCCCTTCCCAACTA
UL51 R	CCTGAGCGGACCGACGCTATTTC
UL35 F	ATGTTTGCGACCTACGACCACG
UL35 R	CGCACAATTGCGCCGATCC
pUL51Exp. F	GGACCTACTACATCTGCCAGCGGAACA
pUL51Exp. R	CGCCCTGCGCCTGAATGCCCAAG
GAPDH F	GGCCTGAACCACGAGAAGTATAA
GAPDH R	CCCTCCACGATGCCAAAGT

To construct the BoHV-1 BAC clone, pMDgB-BAC-UL26 was co-transfected with the wild-type BoHV-1 genome into MDBK cells using the calcium phosphate procedure [25]. Several green fluorescence-positive recombinant viruses (Figure 1C) were plaque purified [25] and analyzed by PCR. The circular, replicative form of vBoHV-1 BAC DNA was extracted from MDBK cells and electroporated into *Escherichia coli* strain GS1783 as previously described [24]. Positive clones were identified by PCR and nucleotide sequencing. One positive clone was designated pBoHV-1 BAC for further use. To delete the BAC sequence containing the *gfp* gene, pBoHV-1 BAC was co-transfected with pCAGGS-NLS/Cre into cultured cells. A virus without the *gfp* gene was obtained by plaque purification and designated vBoHV-1 (wild-type virus).

### Construction of a pUL51 mutant virus

BoHV-1 pUL51 consists of 243 amino acids. The pUL51 mutant was constructed by deleting amino acids 76-232 (Figure 2A, 2B) using two step, λ-red-mediated mutagenesis [27]. To delete specific nucleotides from the UL51 gene, the primers UL51Δ(76-232) F/R (Table 2) were used to amplify the kanamycin resistance gene from the pEPkan-S plasmid, which was provided by Dr. Nikolaus Osterrieder [28]. The PCR product contained the upper and lower homolog arms of the deleted region and a 3′ *I-SceI-aphAI* cassette, and it was purified using the Cycle Pure kit (Omega Bio-Tek, Norcross, GA, USA) according to the manufacturer's instruction, and confirmed by DNA sequencing. The gel-purified, DpnI-digested PCR product was electroporated (1.8 kV, 25 μF capacitance, and 200 Ω resistance) into GS1783 cells that contained pBoHV-1 BAC, and cultured on Luria-Bertani (LB) agar plates containing 30 μg/ml chloramphenicol and 30 μg/ml kanamycin. Several clones were picked, and the first λ-red recombination event was identified using PCR and DNA sequencing. For the second λ-red recombination event, 20 μl of an overnight culture of positive bacteria was added to 2 ml of LB broth containing 30 μg/ml chloramphenicol, and the culture was incubated for 2-3 h at 32°C. Then, 2 ml of LB broth containing 30 μg/ml chloramphenicol and 2% L-arabinose was added. After a 1-h incubation at 32°C, the culture was incubated at42°C for 30 min, with shaking at 200 rpm, and then the culture was shifted to 32°C for 2 h. The culture was plated onto LB agar plates containing 30 μg/ml chloramphenicol and 1% L-arabinose. Positive clones were identified by PCR and DNA sequencing and named pBoHV1-UL51Δ76-232 BAC. The BAC sequence containing the *gfp* gene was deleted using pCAGGS-NLS/Cre, and the resultant virus was designated the vBoHV1-UL51Δ76-232 (UL51 mutant) virus.

### Construction of hemagglutinin (HA)-tagged recombinant viruses

To characterize and find localization of pUL51, an HA tag was also added to the carboxyl terminus of pUL51 in pBoHV-1 BAC (Figure 2C) using primers UL51-HA F/R (Table 2). The HA tag was also added to the minor viral capsid protein pUL35 of pBoHV-1 BAC (Figure 2D) and pBoHV1-UL51Δ76-232 BAC (Figure 2E) using the UL35-HA F/R primers (Table 2) following two-step, λ-red-mediated mutagenesis [27] as previously described. Positive clones were identified by PCR and DNA sequencing. The BAC sequence was deleted by transfecting the respective BAC DNAs with pCAGGS-NLS/Cre into cultured cells.

### Reversion of pBoHV1-UL51Δ76-232

The pBoHV1-UL51Δ76-232 rescued virus was constructed using the two-step, λ-red recombination system [27]. To do so, the deleted region of pUL51 (with BamHI and EcoRV restriction sites) was amplified by LA *Taq* polymerase (Clontech, Mountain View, CA, USA) using primers pCDNA4 51R F/R and cloned into pCDNA4 (Invitrogen, Carlsbad, CA, USA). Then, the *I-SceI-aphAI* cassette was cloned from pEPkan-S using the PstI restriction site. The 5′ end of the forward primer (pCDNA4 51R kana F) contained 50 bp after the cloning site, while the 3′ end contained 24 bp that were complementary to the *I-SceI-aphAI* cassette. The reverse primers (pCDNA4 51R kana R) contained the *I-SceI-aphAI* cassette sequence and the restriction site PstI. This cloned sequence from pCDNA4 was amplified using primers that have 50 bp of sequences that are homologous to the recombination sites on each side of the pUL51 gene. The generated PCR product was then electroporated into electrocompetent GS1783 cells containing pBoHV1-UL51Δ76-232 BAC. Then, the bacteria were plated onto LB agar plates containing 30 μg/ml chloramphenicol and 30 μg/ml kanamycin and grown at 32°C for 24 to 42 h. Positive clones were identified by PCR and DNA sequencing. To obtain the required reversion, 20 μl of an overnight culture of the positive clone containing the *I-SceI-aphAI* kanamycin resistance cassette was added to 2 ml of LB broth containing chloramphenicol. After 2-3 h of growth at 32°C (with shaking), 2 ml of LB broth containing chloramphenicol and L-arabinose was added. After 2-h incubation at 32°C, the culture was shifted to a shaking water bath and incubated at 42°C for 30 min. Then, the culture was shifted to 32°C and grown for 2-3 h. Then, the culture was plated onto LB agar containing chloramphenicol and L-arabinose. Positive clones were verified by PCR and DNA sequencing. The revertant virus was rescued by transfecting pBoHV1-UL51R BAC DNA with pCAGGS-NLS/Cre into cultured cells, and it was designated the vBoHV1-UL51R (revertant) virus.

### Growth curve analysis

For single-step and multi-step growth kinetics, confluent MDBK cells were infected with the indicated viruses at MOIs of 1 and 0.01. After a 1-h incubation at 37°C, viral inocula were removed and non-attached viruses were inactivated by low-pH citrate buffer [29] treatment for 1 min at room temperature. Infected MDBK cells were washed twice and overlaid with fresh cell culture medium. Intracellular and extracellular viruses were harvested at the indicated time intervals [30]. After three freeze-thaw cycles, the virus was subsequently titrated in MDBK cells as described previously [31].

### Plaque assay

Confluent MDBK cells in a 6-well culture plate were infected with the indicated viruses. Each well contained 100 PFU of the indicated virus. After 1-h incubation at 37°C, the viruses were removed, and the wells were overlaid with methylcellulose. After 48 h of incubation at 37°C in a 5% CO_2_ atmosphere, the plaques were fixed with formaldehyde and stained with crystal violet. For each recombinant virus, 50 plaques were measured microscopically in two independent experiments.

### SDS-PAGE and western blotting

Confluent MDBK cells were infected with the indicated viruses. After 2 h of incubation at 37°C, the viral inoculum was removed and fresh cell culture medium was added after washing the cells. After 18 h of incubation, the medium was removed, and the cells were washed with cold 1× phosphate-buffered saline (PBS). The cells were collected in ice-cold PBS with the aid of a cell scraper. Cell suspensions were centrifuged at 160 × g for 10 min at 4°C to obtain the cell pellet. The cell pellet was lysed at 4°C using lysis buffer (50 mM Tris, 1 mM ethylenediaminetetraacetic acid (EDTA), 150 mM NaCl, and 1% Triton X-100) containing a 1× protease inhibitor cocktail (Sigma-Aldrich, Shanghai, China). The proteins were collected after clearing the cell debris. The collected proteins were further processed for SDS-PAGE. After running the gel, the proteins were transferred to nitrocellulose membranes (Millipore, Hong Kong, China). The nitrocellulose membranes were blocked overnight in 5% non-fat milk. Then, the membranes were incubated with either a mouse anti-HA antibody (Beyotime, Haimen, China) at a 1:1000 dilution or β-actin (Beyotime) at 1:1000 dilutions, followed by a horseradish peroxidase-conjugated secondary antibody. Bound antibodies were visualized using the Super Signal West Pico Chemiluminescent Substrate (Thermo Fisher Scientific, Waltham, MA, USA). All membrane washes and antibody dilutions were performed in Tris-buffered saline containing 0.1% Tween-20.

### Silver staining

Silver staining was performed as described previously [32], with slight modifications. Briefly, after SDS-PAGE, the gel was fixed in a fixative solution (40% ethanol, 10% acetic acid, and 50% H_2_O) for 1 h. After fixation, the gel was washed in H_2_O overnight and sensitized with 0.02% sodium thiosulfate for 1 min. Then, the gel was washed three times in H_2_O and incubated with a cold 0.1% silver nitrate solution for 20 min. After a brief rinse in water, the gel was developed in a 3% sodium carbonate and 0.05% formaldehyde solution. After detecting the protein bands, staining was terminated using a 5% acetic acid solution for 5 min.

### Mass spectrometry

Confluent MDBK cells were infected with the vBoHV-1 and vBoHV1-UL51-HA viruses at an MOI of 3. After 18 h, the cells were lysed, and the lysate was incubated with anti-HA-tag monoclonal antibody-conjugated magnetic agarose beads (MBL, Nagoya, Japan) for 6 h at 4°C on a rotator. After four consecutive washes, bound proteins were eluted by boiling the agarose in SDS-PAGE sample buffer. The gel was silver stained and the selected bands were excised and processed by trypsin digestion for matrix-assisted laser desorption/ionization time of flight (MALDI-TOF) mass spectrometry.

### Immunofluorescent confocal microscopy

Freshly grown MDBK cell monolayers on glass coverslips were infected with the indicated viruses. At different time points, the cells were fixed in 4% paraformaldehyde, permeabilized with 0.2% Triton X-100, and blocked with 2% bovine serum albumen and 1% goat serum. After washing, the cells were incubated with mouse anti-HA (1:500 dilution) and rabbit anti GM130 (Abcam, Cambridge, UK) (1:250 dilution) antibodies. After subsequent washing, the cells were stained with Cy3-conjugated goat anti-mouse (1:1,000 dilution) and fluorescein isothiocyanate (FITC)-conjugated goat anti-rabbit (1:1,000 dilution) antibodies. After three washes, nuclei were stained with 4, 6-diamidino-2-phenylindole (DAPI). Then, the coverslips were mounted onto slides using anti-fade mounting medium. Images were examined and captured with a Zeiss LSM 510 laser-scanning confocal microscope (Carl Zeiss, Jena, Germany).

### Transmission electron microscopy (TEM)

MDBK cells were infected with the indicated viruses at an MOI of 3 PFU per cell. After 18 h, the cells were collected and washed by low-speed centrifugation. The cells were fixed with 5% glutaraldehyde in 0.1 M sodium cacodylate buffer at room temperature. Then, the cells were washed and post-fixed with 1% osmium tetraoxide. Subsequently, the cells were rinsed and dehydrated using graded ethanol. Then, the cells were saturated with uranyl acetate, a graded ethanol series, and three changes of propylene oxide, and the samples were embedded, polymerized, and sectioned. The samples were analyzed by a transmission electron microscope (Hitachi 7000FA^®^, Tokyo, Japan). We had counted 40 cells randomly with more than 25 fields each of vBoHV-1 and UL51Δ76-232 infected cells.

### Rabbit infections

Viral infections of rabbits were performed as described elsewhere [25]. Briefly, 24 rabbits were randomly allocated to four groups of six animals each, and each group was housed separately and provided with food and water *ad libitum*. Groups 1, 2, and 3 were infected with the vBoHV-1, vBoHV1-UL51Δ76-232, and vBoHV1-UL51R viruses, respectively, while group 4 was left as an untreated blank control. For infection, rabbits were sedated and then intranasally infected with 2×10^7^ PFU (1×10^7^ PFU per nostril) of the viruses. Nasal swabs were collected during acute and latent infections at 0, 1, 3, 5, 7, 10, 15, 14, 17, and 20 d post-infection (dpi). At 20 dpi, the rabbits in each group were injected with dexamethasone (2.8 mg/kg) for five consecutive days to reactivate the virus from latency. After reactivation, nasal swabs were collected at 26, 27, 28, and 31 dpi. Serum samples were also collected during the acute, latent, and reactivation periods on the designated days. Swab samples were collected in 2 ml of Dulbecco's modified Eagle's medium containing penicillin/streptomycin antibiotics, and stored at −80°C. The animals were slaughtered at 31 dpi to observe pathological changes. For virus quantitation, nasal swabs were thawed at 37°C, and subsequently vortexed and centrifuged at 10621 × g. The supernatant was filtered through 0.22-μm filters, and viral titration was performed using the filtered supernatant from the swab in MDBK cells. The titers of serum neutralizing (SN) antibodies were determined as previously described [25].

### Statistical analysis

The data from the various groups were compared using *t*-tests and two-way analysis of variance (ANOVA) when necessary using Graphpad prism Software.
